# Information-Reduction Ability Assessment in the Context of Complex Problem-Solving

**DOI:** 10.3390/jintelligence13030028

**Published:** 2025-02-26

**Authors:** Xiaoxuan Bu, Huijia Zheng, Xuetao Tian, Fang Luo

**Affiliations:** 1 Faculty of Psychology, Beijing Normal University, Beijing 100875, China; 202131061002@mail.bnu.edu.cn (X.B.); xttian@bnu.edu.cn (X.T.); 2 Peabody College of Education and Human Development, Vanderbilt University, Nashville, TN 37203, USA; huijiazh@umich.edu

**Keywords:** information reduction, computer-based assessment, complex problem-solving, DYNAMIS framework

## Abstract

In this era with an increasing overabundance of information, the ability to distill relevant information, i.e., “information reduction”, is becoming more crucial to daily functioning. However, the fact that information reduction is most prominent in complex situations poses challenges for measuring and quantifying this ability. Existing assessments tend to suffer from either too little complexity, compromising ecological validity, or too much complexity, which makes distinguishing and measuring information-reduction behavior difficult. To address this gap in the literature, our study developed a novel assessment tool, the Little Monster Clinic (LMC), designed to capture the information-reduction process within complex problem-solving scenarios. Following the classic complex problem-solving (CPS) framework, LMC simulates real-world medical situations and provides a sufficiently complex task for assessing information-reduction ability. We recruited 303 students to validate our tool and identified six key indicators for information reduction, which demonstrated a high degree of internal consistency (α = 0.83). Structural validity from the confirmatory factor analysis (CFA) supported a one-factor model of information reduction based on the extracted indicators ($\chi_{2}$ = 14.872, df = 5, $\chi_{2}$/df = 2.774, CFI = 0.989, TLI = 0.967, RMSEA = 0.077, SRMR = 0.024). The significant correlation (*r* = 0.43, *p* < 0.01) between LMC and Genetics Lab demonstrated its criterion-related validity. Furthermore, exploratory analysis highlighted the importance of identifying key relevant information during the process of information reduction. These findings lend support to both the theoretical foundation and practical applications of information-reduction assessment.

## 1. Introduction

The concept of “information overload” describes a scenario where individuals are confronted with significantly more information than they can effectively process. An overabundance of information overloads people’s cognitive processing capacities, negatively impacting various aspects such as well-being, problem-solving performance, and decision quality (Che et al. 2019). With the rapid development of digital technology, the amount of information is growing exponentially every day, while human cognitive capacity remains relatively constant. Given these circumstances, the ability to reduce information, that is, selectively processing task-relevant information while filtering out irrelevant details, thereby optimizing cognitive resources, has become imperative for efficient daily functioning (Haider and Frensch 1996). In the realm of cognitive psychology, the concept of information reduction plays a pivotal role in understanding how individuals navigate complex environments. However, despite its importance, information reduction during complex scenarios has yet to be fully explored, due to limitations in assessment tools in terms of task form and complexity.

To address these limitations, we developed a novel complex problem-solving (CPS) assessment tool named Little Monster Clinic (LMC), designed to simulate and capture individuals’ information-reduction processes. Unlike traditional tasks such as the alphabet verification task (AVT)) (Haider and Frensch 1996), which only involve simple stimulus-response paradigms, LMC incorporates multiple exogenous and endogenous variables. It follows a formal DYNAMIS framework (Funke 1992), reflecting the complexity of real-world problems. We examined the psychometric properties of the developed LMC assessment tool; an exploratory analysis highlighted the importance of identifying key relevant information during the process of information reduction.

## 2. Background

### 2.1. Overview of Information Reduction

Information reduction, as its name suggests, refers to the process where information relevant to the task of concern is processed selectively, while irrelevant information is filtered out (Haider and Frensch 1996). Through information reduction, input information is condensed into a less complex output, allowing for more efficient utilization of information in subsequent behaviors. This ability plays an important role in coordinating limited cognitive resources when coping with complexity (Fischer et al. 2012), which comprises a critical step in a range of processes. For instance, in problem-solving, information reduction is used to abstract from irrelevant information, so that more efficient planning could be achieved in the resulting smaller problem space (Fischer et al. 2012; Newell and Simon 1972). In skill acquisition, information-reduction behavior serves as a critical distinction between experts and novices, as experts learn to process information more efficiently by paying more attention to task-relevant information (Haider and Frensch 1999; Ruf et al. 2023). Some theories have further identified information-reduction ability as a prerequisite for behavior in general (Gibson 1979; Lee and Anderson 2001). However, contrary to its critical role in behavior, assessment tools for general information-reduction ability are still underdeveloped.

While the role of information reduction has been implicated in many disciplines (e.g., psychology (Dreisbach and Haider 2008; Haider and Frensch 1999; Soetens 1998), sports (Abernethy 1993; Helsen and Pauwels 1993), driving (Underwood et al. 2002), air traffic control (Niessen et al. 1999), etc.), these works primarily focused on simple cognitive tasks or focused on the skills of experts and novices in specific domains, lacking research on general abilities to deal with complex situations. The primary reason for this phenomenon is that the process of information reduction inherently involves an implicit mode of information processing. This makes it particularly challenging to directly manifest through the explicit actions taken by individuals when they are engaged in solving complex problems, thereby posing difficulty in developing explicit assessment methods. Existing assessment approaches typically use simple stimulus-response tasks, involving the reduction of only one or two variables at a time, e.g., the alphabet verification task (AVT) used in Haider and Frensch (1996), which asked participants to extract relevant letters from an alphanumeric string and leveraged response time to indicate whether the information-reduction process had occurred. Since such a task only involves simple information stimuli, it cannot represent the relevant/irrelevant relationships among variables, which are fundamental to basic information reduction in complex scenarios (Garner 1962; Posner 1964). Therefore, the setup of these tasks does not sufficiently mirror the complexities of real-world scenarios, meaning that their results may be inconsistent with real-world performance.

Without an appropriate assessment tool for information-reduction ability, we are missing a perspective on a critical step in complex cognition. Thus, we argue that an effective assessment tool for information-reduction ability is necessary to push related theoretical work in multiple disciplines to a more concrete level. The current study seeks to develop a new assessment tool that addresses this matter.

### 2.2. Information Reduction in Complex Problem-Solving

Information reduction is a significant dimension in complex problem-solving (CPS) (Fischer et al. 2012). The theory of information reduction in the context of CPS was first proposed by Klauer (1993), who identified the necessity of simplifying complex problem situations into concise representations during problem-solving. Gaschler (2009) elaborated on the following: “Information Reduction applies in situations in which tasks contain both relevant and irrelevant information, and denotes a change from a strategy involving processing all elements of a task to a processing-relevant-elements-only strategy”. Relatedly, Fischer et al. (2012) indicated in their research that “omitting irrelevant task components and finding a parsimonious representation of the problem may enable and foster the search for a solution to a complex problem”. It is clear that in CPS, an individual’s ability to identify key variables as well as relationships and disregard irrelevant ones is crucial to internal problem representation and problem-solving performance.

Compared to these theoretical involvements, empirical research on information reduction in the context of CPS has made less progress. Current CPS assessment tools can be primarily divided into two categories: more ecologically focused, computer-simulated microworlds and multiple complex systems (MCSs) constructed based on formal frameworks. Although these microworlds offer a closer approximation to real-life scenarios, such as the “Lohhausen” task (Dörner and Wearing 1995), most of these microworlds do not have optimal solution paths and best interventions. Researchers could never be entirely certain whether one subject’s solution to a problem was truly better or worse than that of other subjects. Performance-based expert scores are always not comparable, as demonstrated by Funke (1992). Furthermore, Funke (1992, 1993) introduced a new microworld framework, DYNAMIS, which enables experimenters to implement various types of simulated systems that all have one formal background in common. Within this framework, the problem-solving scenarios consist of several exogenous variables (which can be manipulated by the test taker) and several endogenous variables (which are connected to the exogenous and/or endogenous variables by linear equations and cannot be manipulated directly). The process for these tasks usually comprises two primary phases: (a) knowledge acquisition and (b) knowledge application. Genetics Lab is a representative tool that follows this framework (Sonnleitner et al. 2012). Then, on the basis of the formal frameworks proposed by Funke (2001), Greiff et al. (2012) introduced the MCS approach, in which underlying system structures can be linear structural equations (LSEs) or finite state automata (FSA). Current multiple complex systems such as MicroDYN and Genetics Lab were developed based on the LSE framework, while the FSA framework gave rise to the MicroFIN.

These two types of assessment tools face different challenges in measuring information reduction. Microworlds tend to involve highly complex system structures that can, in theory, simulate real-life situations and realistically activate individuals’ information reduction abilities. However, in the context of such highly complex tasks, every operation performed by participants as they strive to reach the goal state is affected by a multitude of interrelated cognitive mechanisms. Consequently, it becomes particularly challenging to identify specific indicators that can effectively evaluate the process of information reduction. On the other hand, as it is necessary for multiple complex systems constructed based on formal frameworks (e.g., MicroDYN) to maintain consistency across different scenarios, the complexity of problem scenarios is greatly reduced (Greiff et al. 2015; Schoppek and Fischer 2015). Even without information reduction, participants may still achieve their goals smoothly, meaning that these tasks tend to be insufficient for activating information reduction in individuals. Additionally, classic DYNAMIS tasks are usually divided into “knowledge acquisition” and “knowledge application” phases (Greiff et al. 2012). This existing two-phase framework does not allow enough space for reflecting the cognitive process of information reduction. Possibly due to the difficulties identified above, commonly used assessment tools in CPS often do not include information reduction as a dimension, despite its crucial involvement in the problem-solving process. In this study, we seek to address these difficulties in a new DYNAMIS-based assessment tool, tailored for assessing information-reduction ability.

### 2.3. The Present Study

Our assessment task is designed to involve multiple task-relevant/irrelevant variables. Participants’ ability to selectively process these variables, i.e., information-reduction ability, is directly reflected in their operation process, as represented by whether they trigger irrelevant variables and discover key variables during the task. We aim to utilize these parameters as a quantifiable measure of participants’ information-reduction ability and further validate the assessment’s usability through reliability and validity analyses.

## 3. Design

### 3.1. Assessment Task Overview

The CPS assessment tool developed in this study is a computer-based system called the “Little Monster Clinic (LMC)”^1^, which expands upon the widespread formal DYNAMIS framework. This tool seeks to simulate real-world problem-solving by presenting a medical scenario. To avoid the influence of prior knowledge, the semantic embedding of the LMC is entirely fictive, which means that the requirement for prior knowledge is very low. For example, participants are prompted to treat medical symptoms displayed on a little monster from an alien planet. Additionally, the variable relationships between “physiological indicators” and “symptoms” were not designed to mirror real-world situations. For instance, in our scenario, the “mild fever” could result in changes to the “head shape”, a phenomenon that has no basis in reality. Participants are expected to utilize the designed relationship between symptoms that the little monster displays, their physiological indicators, and the medication provided. For instance, in the assessment task, participants can adjust the dosages of various medications to control the little monster’s physiological indicators, with the ultimate goal of restoring the little monster to health.

**Figure 1:**
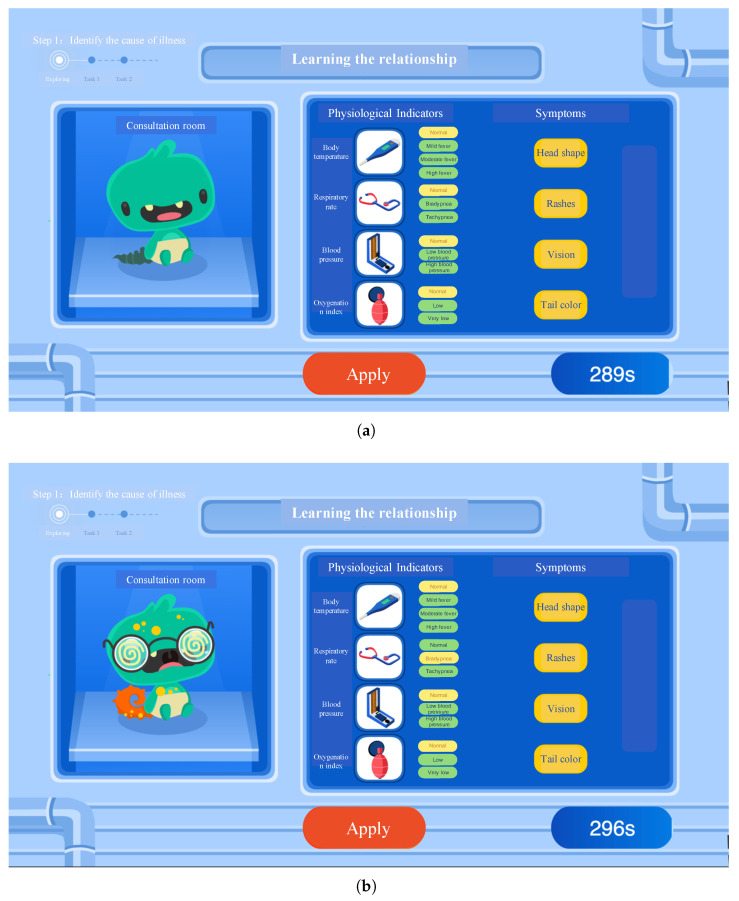
Schematic diagram of the first phase in the information-reduction step. (**a**) The little monster starts in a healthy state. (**b**) The little monster has an illness with “Respiratory rate” being “Bradypnea”.

**Figure 2:**
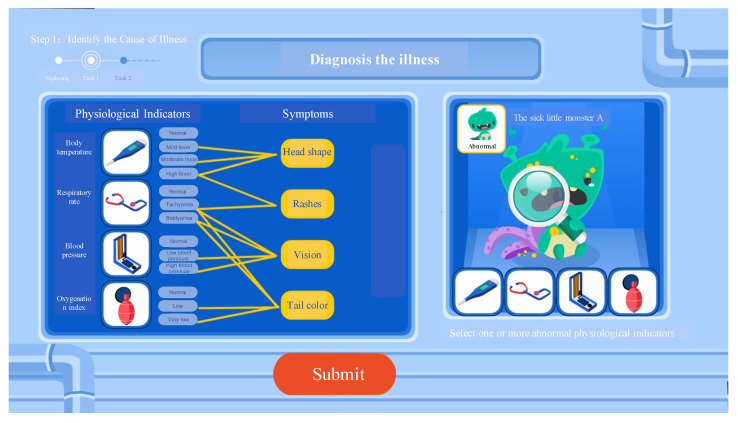
Schematic diagram of the second phase in the information-reduction step.

This assessment tool expands and innovates upon the five dimensions of the classic CPS assessment framework (Fischer et al. 2012), and designs three steps to assess participants’ CPS abilities: identifying the cause of illness, learning how to administer medication, diagnosing and providing treatment. Among these, the first step specifically assesses information-reduction ability, which is the focus of this paper. As above-mentioned, the classic DYNAMIS task framework includes two phases, namely, knowledge acquisition and knowledge application. The knowledge acquisition phase is further divided into the following two processes: (1) problem exploration, and (2) problem representation, where participants collect and process information about the task scenario (Fischer et al. 2012; Molnár and Csapó 2018). The collected information is then applied to problem-solving, which is the theme of the knowledge application phase. The process to assess information-reduction ability includes two phases, similar to the DYNAMIS task: Phase 1 (knowledge acquisition, see Figure 1) instructs participants to explore the relationship between the little monster’s symptoms and physiological indicators (the problem exploration process), and then submit a relational diagram based on their findings (the problem representation process). In Phase 2 (knowledge application, see Figure 2), after the participant submits their own relational diagram, they will be provided with a relational diagram showing the correct relationships between the variables. Then, participants are required to determine which physiological indicators are associated with the current symptoms of the small monster, based on the correct relationship diagram provided by the system. If there is uncertainty regarding the relevance of certain physiological indicators, participants can perform checks to confirm their relationship. Phase 2 contains two of these diagnosis tasks, which are identical except for the little monster’s symptoms.

### 3.2. Activating Information Reduction

As previously noted, information-reduction ability in complex problem situations primarily involves selectively processing task-relevant variables and ignoring irrelevant ones. In this study, we refer to Fischer et al. (2012) and achieve task complexity by increasing the number of variables involved in the task, as well as increasing the complexity of the relationships between them.

**Figure 3:**
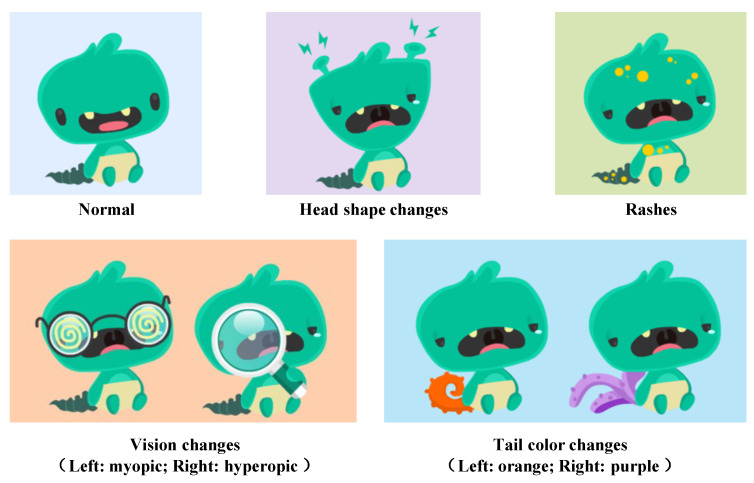
Illustration of little monster’s symptoms.

In terms of the number of variables, the assessment task includes four exogenous variables (physiological indicators) and four endogenous variables (symptoms). Physiological indicator variables include body temperature, respiratory rate, blood pressure, and oxygenation index, each with two levels, ‘normal’ or ‘abnormal’. When these variables are ‘normal’, symptoms will also disappear. ‘Abnormal’ indicators are further divided into 3–4 degrees, totaling 13 levels, such as four body temperature levels (normal, mild fever, moderate fever, high fever), three respiratory rate levels (normal, bradypnea, tachypnea), three blood pressure levels (normal, low blood pressure, high blood pressure), and three oxygenation index levels (normal, low, very low). Symptom variables include head shape (normal: square; abnormal: triangular), presence of rashes (normal: no rash; abnormal: rashes), vision (normal: no visual issues; abnormal: myopic or hyperopic), and tail color (normal: green; abnormal: purple or orange), as illustrated in Figure 3.

**Figure 4:**
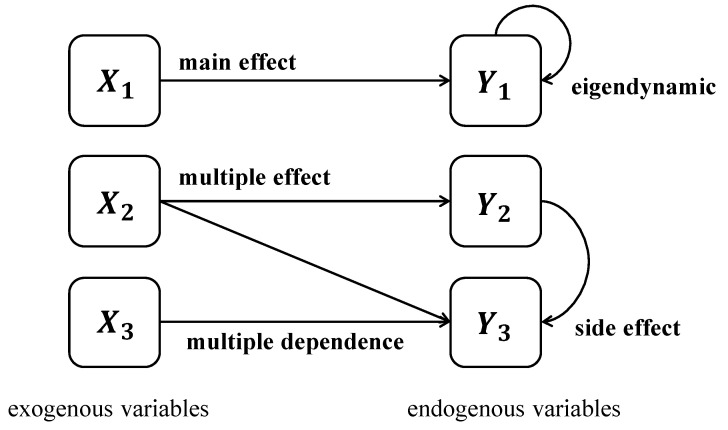
Schematic diagram of possible relationships between variables.

**Figure 5:**
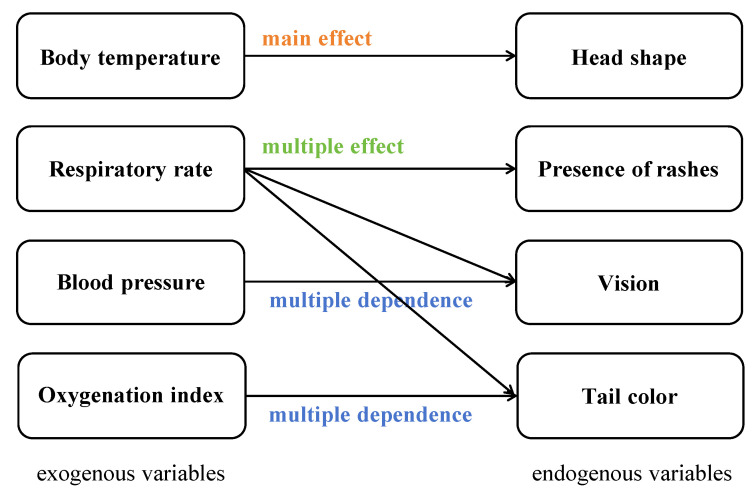
The relationship between physiological indicators and symptoms in the “Little Monster Clinic”.

In terms of increasing the complexity of relationships between variables, we refer to Greiff and Funke (2009)’s idea on the relationships between variables in DYNAMIS. As shown in Figure 4, there are three possible relationships between exogenous and endogenous variables, as follows: (1) main effect, referring to the causal relations from a single exogenous variable to a single endogenous variable; (2) multiple effects, referring to an exogenous variable being involved in more than one main effect; and (3) multiple dependence, referring to the effects on an endogenous variable being influenced by more than one exogenous variable. We employ all of these in our assessment. As Figure 5 displays, head shape has a main effect on body temperature; respiratory rate has multiple effects on rashes, vision, and tail color; vision exhibits multi-dependence with respiratory rate and blood pressure; and tail color also demonstrates a multiple-dependence relationship with respiratory rate and oxygenation index. These different relationships implicate the variables’ varying degrees of relevance for problem-solving during the task. Therefore, participants are required to identify which relationships are more crucial to the task at hand, i.e., perform information reduction regarding the variable relationships. Notably, in our study, we focus on information-reduction ability, which involves identifying the irrelevance and importance of exogenous variables based on endogenous variables within a relatively short timeframe. This cognitive process is inherently different from capturing the long-term effects. Therefore, we did not consider “eigendynamics” and “side effect”, which involve interactions between endogenous variables over time, as these were beyond the scope of our investigation into information reduction.

To summarize, over the assessment process, in Phase 1, participants construct cognitive representations of the relationships between physiological indicators and symptoms by controlling the exogenous variables and observing the resulting changes in endogenous variables. In Phase 2, participants infer the exogenous variable by observing the changes in endogenous variables. In other words, participants first need to refer to the correct relational diagram between physiological indicators and symptoms, to identify abnormal physiological indicators. Then, for the physiological indicators that remain unclear, participants are instructed to find the abnormal indicators with minimal inspection steps. Finally, participants submit their answers. This task process is designed to necessitate information-reduction ability, as in understanding the varying relevance of variables, as well as the relationships between them.

## 4. Methods

### 4.1. External Measures

**Genetics Lab**: This study used the Genetics Lab tool (Sonnleitner et al. 2012) as a measure of external validity. Genetics Lab is a computer-based complex-system assessment tool used for evaluating complex problem-solving abilities, consisting of 12 scenarios. It has been tested for reliability and validity, with good acceptance among students (Sonnleitner et al. 2012), which makes it suitable as a reference for validating this new assessment tool. Primary scores of Genetics Lab include a process-oriented score indicating participants’ efficiency when exploring new problem situations (systematic exploration), a score evaluating participants’ graph of relational knowledge between genes and characteristics (system knowledge), and another process-oriented score reflecting participants’ goal achievement (control performance).

**Personal Information and Task Experience Questionnaire**: Considering the game-based format and the medical context of the assessment tasks, performance may be influenced by participants’ personal experiences in related fields. To account for this in our study, we administered a personal information and task experience questionnaire to collect participants’ demographic information, experience during the task, and previous medical knowledge. This questionnaire contains 21 items, distributed as follows: demographic information (9 items, including ID, school, grade, age, gender, whether parents work in medical occupations, frequency of hospital visits, and time spent playing computer games); task experience (10 items on a 5-point Likert scale, from 1 (strongly disagree) to 5 (strongly agree)), measuring the participants’ perception of the task’s complexity, visual appeal and enjoyability; and previous medical knowledge (2 items), measuring the participants’ medical knowledge, such as the ability to recognize antibiotics. Please refer to Supplementary Materials for specific questionnaire content.

### 4.2. Participants and Procedure

A total of 303 university students were recruited as participants in two groups: 37 (male: 40.5%; female: 59.5%; age: 21.51 (±0.79)) in the first group, and 266 (male: 32.3%; female: 67.7%; from first- and second-year graduate students) in the second group. All participants in these two groups were required to complete the LMC assessment. This experiment session, which lasts approximately 1 h in length, includes instructions and a practice session to ensure understanding of the procedures. Moreover, the first group completed an additional Genetics Lab, and a personal information and task experience questionnaire to verify validity. Before the data collection, we conducted an a priori power analysis to ensure that our sample size was adequate to detect the expected effect size with sufficient statistical power (Faul et al. 2007). Using the pwr package in R3.4.2, specifically the pwr.r.test() function, we calculated the minimum required sample size based on an anticipated correlation coefficient (*r*) of 0.5, a desired power level of 0.8, and a significance level (α) set at 0.05 for a two-sided test. The results indicated that approximately 28.25 participants would be needed. Therefore, 37 participants met the validation requirements. All assessment data for this group took approximately one week to collect. All tests were administered by the same researcher to ensure consistency. Each participant completed their assessments within the same day, in a quiet and tidy environment. After signing the consent form, participants were introduced to the procedures of the experiment. To minimize the order effect, we tested this group under a balanced design, with 19 participants taking LMC first and 18 taking Genetics Lab first. All first-group participants completed the personal information and task experience questionnaire after the LMC assessment.

### 4.3. Scores

The first step of LMC assesses information reduction based on the participants’ ability to selectively process relevant variables and ignore irrelevant ones, in the context of the task, when checking physiological indicators. In general, for an optimal strategy considering the three types of relationships between exogenous and endogenous variables identified before, the approach to each type varies, as follows: variables involved in the main effects (a single exogenous variable influences a single endogenous variable) are easily identified and can be treated as irrelevant with no checking; variables with multiple effects (influencing multiple endogenous variables) should be prioritized for checking due to their significant impact on problem-solving; while variables with multiple dependencies (influenced by multiple exogenous variables) should be checked after those with multiple effects have been identified.

For instance, in Task 1 of Phase 2 (see Figure 2), the little monster’s symptoms include head shape change, rashes, hyperopia, and tail color change. Here, body temperature must be abnormal, since head shape is relevant only to it. So body temperature could be identified as an irrelevant variable with no checking. The key variable is respiratory rate, as both vision and color changes are related to it. More specifically, since vision is related to respiratory rate and blood pressure, and tail color is related to respiratory rate and oxygenation index, it could be inferred that—on this occasion—if respiratory rates are normal, blood pressure and oxygenation index must be abnormal; while if respiratory rates are abnormal, the other two variables are left uncertain. Therefore, the respiratory rate should be prioritized for checking. The types of variables that were checked, and the order in which they were checked, compared to the optimal strategy, could reflect participants’ efficiency in information reduction and, thus, serve as the LMC’s evaluation criteria for assessing information-reduction ability.

According to these criteria, six key indicators are extracted from the two tasks in Phase 2. Specifically, for each task, the indicators assess whether participants were able to recognize that variables vary in their degree of relevance, i.e., not checking indicators in the default order; whether participants were able to identify relevant variables, i.e., prioritize checking respiratory rate; and whether participants were able to ignore irrelevant variables, i.e., ignore body temperature. In the end, a total of six indicators are scored on a 0–1 (0/1 for No/Yes) scale, with a maximum score of 6 (Table 1).

Additionally, the efficiency of exploration and accuracy of the relational diagram generated during Phase 1 are considered to be related to information-reduction ability. Thus, we extract the following two indicators from Phase 1: the exploration efficiency score and the problem representation score. Efficient exploration is scored according to the proportion of efficient steps among total steps, as defined by the VOTAT strategy (Tschirgi 1980), where multiple identical steps will only be calculated once, since repeated steps cannot generate any additional useful information. The VOTAT strategy is a representative systematic strategy that, as its name suggests, “varies one thing at a time” to identify the effects of independent variables on dependent variables, allowing the problem solver to “coherently infer the consequences of single interactions” and “build viable structural knowledge about parts of the system structure” (Fischer et al. 2012; Vollmeyer et al. 1996). Here, we choose this strategy as the sole criterion for efficiency during exploration. Relational diagram answers are scored with a set of criteria equivalent to Genetics Lab, which refers to the scoring method proposed by Funke (1992). The score is calculated according to Equation (1).

(1) Rel=(1−Prel)×(Crel/Cmax)−Prel×(Erel/Emax),

where *Prel*, set as 0.5, denotes the probability that the answer was guessed, *Crel* denotes the number of correct lines in the participant-generated diagram, *Cmax* denotes the maximum number of correct lines possible, *Erel* denotes the number of incorrect lines in the participant-generated diagram, and *Emax* denotes the maximum number of incorrect lines possible.

## 5. Results

### 5.1. Acceptance of LMC Among Test Takers

Among the data collected, a total of 37 personal information and task experience questionnaires were valid for analysis. Among these, the score for the enjoyability of the task was 4.36 (±0.64), with 81.1% of individuals expressing agreement or strong agreement; in terms of problem-solving design, 86.5% believed that the task reflected their complex problem-solving ability, resulting in a score of 3.92 (±0.98), 94.6% reported feeling that the task was somewhat difficult, returning a score of 4.47 (±0.63), and 100% reported that the task required full attention and careful consideration. These responses suggest that the task was sufficiently engaging and challenging to motivate problem-solving for most participants, making it suitable for application in more practical assessment contexts.

Taking into account the special context of the task, the personal information questionnaire also included questions to assess participants’ prior medical knowledge. Statistical analysis showed that there is a significant correlation between the two items (*r* = 0.75, *p* < 0.01). And both items showed no significant correlation to their performance on the LMC test, including exploration efficiency (*r* = 0.07, *p* = 0.68 and *r* = 0.05, *p* = 0.76), problem representation (*r* = 0.03, *p* = 0.88 and *r* = 0.08, *p* = 0.66), and information reduction (*r* = −0.18, *p* = 0.30 and *r* = −0.18, *p* = 0.30), suggesting that the task design was sufficient to evade confounds from prior medical knowledge.

### 5.2. Descriptive Statistics

This study analyzed the performance of 303 participants across 6 information-reduction indicators. As shown in Table 2, more than 50% of the participants demonstrated some kind of strategy use by not following the default order when examining physiological indicators in both tasks. However, less than 25% of participants were able to fulfill the indicator for checking the respiratory rate in both tasks in Phase 2, indicating that few individuals were able to clearly identify and prioritize key relevant variables during problem-solving. In comparison, participants performed slightly better in ignoring irrelevant variables, i.e., not checking body temperature, with over 30% of participants fulfilling this indicator in both tasks. These outcomes indicate that identifying key relevant variables may be more difficult than ignoring irrelevant variables, requiring a higher level of information-reduction ability.

### 5.3. Psychometric Properties

Data from 303 participants were analyzed to assess the internal consistency of the information-reduction indicators, giving Cronbach’s α = 0.83 and coefficient ω = 0.82 (Flora 2020). Table 3 gives the discrimination indices for each indicator, all of which are above 0.50, indicating their high discriminative effect. Correlation analysis presented in Table 4 also reveals significant relationships across all indicators.

This study also analyzed the correlation between learning performance in Phase 1 (reflected by the exploration efficiency score and problem representation score), and fulfillment of information-reduction indicators in Phase 2. As shown in Table 5, significant correlations were found between the fulfillment of information-reduction indicators, overall information-reduction score, and exploration efficiency and problem representation scores. As different aspects of CPS ability, information reduction has a certain inherent correlation with others.

**Figure 6:**
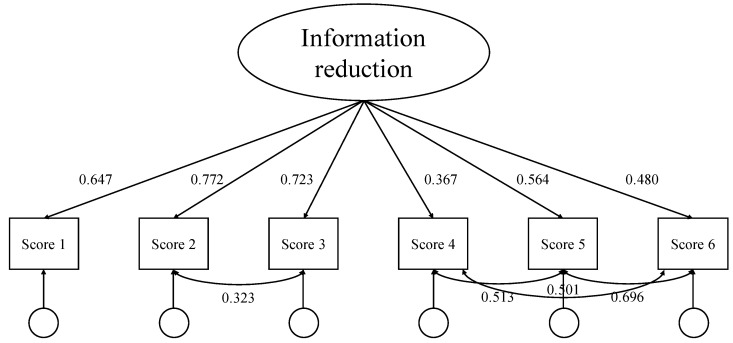
Confirmatory factor analysis (CFA) model on information-reduction ability assessment.

Confirmatory factor analysis (CFA) was conducted using R 3.4.2 to examine the structural validity of the LMC assessment tool. The results indicated that the one-factor model fit the data well ($\chi_{2}$ = 14.872, df = 5, $\chi_{2}$/df = 2.774, CFI = 0.989, TLI = 0.967, RMSEA = 0.077, SRMR = 0.024), as shown in Figure 6. The standardized factor loadings for information reduction ranged from 0.367 to 0.772. The comparative fit index (CFI) and Tucker–Lewis index (TLI) were both above 0.95, suggesting an excellent fit. The root mean square error of approximation (RMSEA) and the standardized root mean square residual (SRMR) were well below the recommended cutoff of 0.08, indicating a good model fit.

As for criterion-related validity, this study used Genetics Lab as an external validity criterion for the information-reduction assessment tool and evaluated participants using the scoring program written for Genetics Lab with R3.4.2 (Keller and Sonnleitner 2012). In this assessment, participants’ average scores for systematic exploration, system knowledge, and control performance were 0.45 (±0.10), 0.82 (±0.15), and 2.36 (±0.46), respectively. The internal consistency values for the systematic exploration score, system knowledge score, and control performance score were 0.93, 0.79, and 0.87, respectively. These results indicate that the Genetics Lab demonstrated excellent reliability. Criterion-related validity was testified by correlating the information-reduction scores from the LMC task to the scores from the Genetics Lab task, yielding a significant correlation (*r* = 0.43, *p* < 0.01) between information-reduction performance in LMC and the system knowledge score in Genetics Lab, indicating good criterion-related validity. Such a result provides robust evidence for the validity of this information-reduction ability assessment.

### 5.4. Differences in Participant Performance During the Two Phases

**Figure 7:**
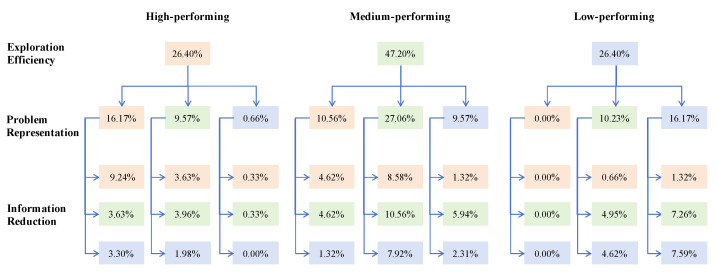
The distribution of participants in three groups during the three processes (orange represents the high-performance group, green represents the medium-performing group, and blue represents the low-performing group).

To further gain perspective on how well the assessment tool distinguishes between ability differences, we performed a group study on participants’ performance during the two task phases. We ranked participants based on their scores in each learning performance indicator, that is, exploration efficiency, problem representation, and information reduction, respectively, with the top 27% of each learning performance indicator classified as high-performance groups, and the bottom 27% classified as low-performance groups. Due to the presence of multiple critical scores, we used the ratio closest to 27% as the grouping criterion (Kelley 1939). As shown in Figure 7, participants with lower performance in exploration efficiency also tended to have lower performance in both problem representation and information reduction. Similarly, participants with high-performance scores in exploration efficiency were more likely to be in the high-performance group for problem representation and information reduction, indicating that participants’ performance in the exploration process during Phase 1 significantly impacted their performance in Phase 2.

We also analyzed performance differences with respect to the following three task strategies relevant to information reduction: not following default order, prioritizing respiratory rate, and ignoring body temperature. For each strategy, we summed performance on the two tasks into one score, with a minimum of 0 and a maximum of 2. Then, we divided the participants into three groups (low, medium, and high) based on their scores (0, 1, or 2). The proportion of participants in each group for each strategy is shown in Table 6. Among the three strategies, prioritizing respiratory rate had the lowest proportion of high performers (12.21%), while not following default order had the highest proportion (39.93%). This may indicate that during the information-reduction process, identifying and prioritizing key relevant variables, represented by prioritizing respiratory rate, is a more challenging strategy to execute.

As shown in Table 7, we also found that participants in the high-performance group, prioritizing respiratory rate, were more likely to be in the high-performance groups for exploration efficiency and problem representation as well. For the strategy of not following default order, participants in the high-performance group are more likely to be in the low-performance groups for exploration efficiency and problem representation. This seems to imply that participants who perform well in exploration efficiency and problem representation are more likely to be good at identifying and prioritizing key variables in complex problems. Also, the findings indicate that, although there is a significant connection between information reduction and problem representation, they do require the use of different ability aspects of CPS.

## 6. Discussions

In this study, we aimed to assess information-reduction ability by leveraging the formal DYNAMIS framework from the complex problem-solving (CPS) literature. This approach allowed us to manipulate the complexity of problem-solving tasks through the relationships between variables involved, addressing previously identified limitations in existing simple stimulus-response tasks. The innovative aspect of our method is particularly evident in the knowledge application phase of the assessment task, where participants evaluate the irrelevance and importance of variables, showcasing an information-reduction process that our tool is uniquely capable of capturing. By doing so, we have not only enhanced the understanding of how individuals process and simplify complex information but also provided an effective assessment tool for information-reduction ability.

### 6.1. The Validity of the Developed LMC Assessment Tool

Following the formal DYNAMIS framework, we developed a novel assessment tool, LMC, for information-reduction ability. In validating our assessment tool, six key indicators of information reduction were extracted, with high consistency and reliability. We evaluated the validity of the LMC tool from the following three aspects: the correlations between CPS dimensions, the structural validity from CFA results, and the criterion-related validity between LMC and Genetics Lab. Firstly, within LMC, there are significant correlations between information reduction and exploration efficiency as well as problem representation, which is consistent with their theory that they are both sub-dimensions of CPS (Fischer et al. 2012). Secondly, CFA results provided evidence supporting the one-factor model of information reduction based on the extracted indicators. Finally, we tested the criterion-related validity of the assessment by examining its consistency with the Genetics Lab assessment and found a significant correlation between it and the system knowledge score in the Genetics Lab assessment. Although system knowledge focuses on the storage and representation of information, information reduction focuses on how to reduce the complexity of information and maintain its effectiveness through information processing. Moreover, both involve the processing of obtained information.

### 6.2. Task Complexity for Information Reduction

Information reduction is the most important aspect of coping with complexity. Increasing the complexity of the information can more effectively stimulate the process of information reduction. By utilizing a formal DYNAMIS framework from CPS literature, we were able to manipulate the complexity of the problem-solving task. Following the Funke et al. (2017)’s idea that simply measuring the ability to solve complex problems by increasing the number of simple problems is not equivalent to solving complex problems in the true sense, we did not design too many questions during the knowledge application phase of LMC, where there are two diagnosis tasks, but focused on the process indicators that reflect information-reduction ability. Although LMC is similar in (formal) structure to MicroDYN and Genetics Lab, it includes four exogenous variables and four endogenous variables, while most MicroDYN tools and Genetics Labs contain two to three. When the number of variables increases, the task complexity will increase non-linearly (Beckmann and Goode 2017; Molnár and Greiff 2023). For example, when considering the number of exogenous variables selected for operation, the task complexity increases exponentially due to the possibility of operating multiple variables simultaneously, i.e., the number of subsets of *n* exogenous variables is given by $2^{n}-1$. A set of 3 variables results in 7 subsets, while a set of 4 variables results in 15 subsets. When further considering the order of operating the selected exogenous variables, the complexity increases at both the exponential and factorial levels, i.e., the sum of the factorial of the subset size of *n* exogenous variables is given by $\sum_{k=1}^{n}C_{n}^{k}\times k!$. A set of 3 variables has 15 ordered subsets, while a set of 4 variables has 64 subsets. The significant increase in complexity imposes a greater cognitive load on the participants, thereby intensifying their problem-solving burden. To resolve problems more swiftly and effectively, it is necessary to simplify the information. Therefore, LMC provides sufficient complexity to assess information-reduction ability.

### 6.3. Key Component of Information Reduction

Since the information-reduction hypothesis was proposed by Haider and Frensch (1996), numerous studies have demonstrated the key processes or strategies of information reduction, including ignoring redundant or unimportant information and identifying key relevant information (Gaschler 2009; Haider and Frensch 1999). However, these studies did not further investigate the importance of these two strategies or their relationship with other cognitive processes (Fischer et al. 2012; Ruf et al. 2023). While some studies have explored the correlation between different phases of CPS, they have often overlooked the process of information reduction (Molnár and Greiff 2023). In this paper, with the goal of assessing individual abilities, we explored three information-reduction strategies: not following default order, which could represent the activation of information-reduction ability, ignoring redundant or unimportant information, and identifying key relevant information. Among these strategies, we found that identifying key relevant information is the most difficult one. Also, participants who scored high in exploration efficiency and problem representation were more likely to achieve high scores in this strategy. Such a finding indicates that the ability to identify key relevant information is at the heart of effective information reduction. Future talent selection and training program designs should focus more on this aspect.

### 6.4. Limitations and Future Research

Some limitations should be taken into consideration in future studies. Firstly, all the samples used to validate this assessment are from undergraduate and graduate students, which may not be sufficiently representative of the general population. Future studies could consider applying this assessment to more diverse populations, or inspect cross-cultural generalizations, to further testify its validity. Secondly, our study focuses on assessing information-reduction ability in a short-term time, which involves making decisions based on immediately available information. The design of LMC does not incorporate “eigendynamics” and “side effect” as shown in Figure 4, which are essential for understanding the self-evolving characteristics of systems over time. This approach may limit our ability to fully capture the dynamic and evolving nature of complex problems. Future research could extend our current framework by incorporating “eigendynamics” and “side effect” to establish a more complex and dynamic task for information-reduction assessment in complex problem-solving. Thirdly, one critical limitation of our study involves the relatively small sample size on criterion-related validity. Although we conducted an a priori power analysis, which indicated that this sample size was sufficient to detect the expected effect size with adequate statistical power, it is important to acknowledge that larger samples generally provide more robust and generalizable results. Fortunately, we ensured that LMC has a high degree of internal consistency, reliability, and construct validity. Future studies with larger sample sizes could be able to refine these estimates and test the replicability of our results. Lastly, information-reduction ability is an important construct, not only in complex problem-solving but also in many other areas. Future studies should expand the scope and explore the role of information reduction in more educational or workplace outcomes.

## 7. Conclusions

This study developed a new assessment tool specifically designed to assess information-reduction abilities within the context of complex problem-solving. The tool followed a formal DYNAMIS framework and incorporated a situational design with higher complexity. Our findings support its psychometric properties in undergraduate and graduate students and highlight the importance of identifying key relevant information in information-reduction ability. Despite certain limitations, including sample size and the dynamic nature of complex problems, our study provides valuable insights into both the theoretical foundation and practical applications of information-reduction assessment.

## Figures and Tables

**Table 1:** Information-reduction indicators.

No.	Indicators
1	Not following default order, Task 1 (Score 1)
2	Prioritizing respiratory rate, Task 1 (Score 2)
3	Ignoring body temperature, Task 1 (Score 3)
4	Not following default order, Task 2 (Score 4)
5	Prioritizing respiratory rate, Task 2 (Score 5)
6	Ignoring body temperature, Task 2 (Score 6)

**Table 2:** Descriptive statistics of information-reduction indicators.

Indicator	Completed Number	Completed Rate
Not following default order, Task 1 (Score 1)	181	59.70%
Prioritizing respiratory rate, Task 1 (Score 2)	71	23.40%
Ignoring body temperature, Task 1 (Score 3)	97	32.00%
Not following default order, Task 2 (Score 4)	155	51.20%
Prioritizing respiratory rate, Task 2 (Score 5)	80	26.40%
Ignoring body temperature, Task 2 (Score 6)	117	38.60%

**Table 3:** Discrimination indices of each indicator.

Indicator	Discrimination
Not following default order, Task 1 (Score 1)	0.54 **
Prioritizing respiratory rate, Task 1 (Score 2)	0.56 **
Ignoring body temperature, Task 1 (Score 3)	0.60 **
Not following default order, Task 2 (Score 4)	0.65 **
Prioritizing respiratory rate, Task 2 (Score 5)	0.54 **
Ignoring body temperature, Task 2 (Score 6)	0.72 **

** p<0.01.

**Table 4:** Correlation analysis among indicators.

	Score 1	Score 2	Score 3	Score 4	Score 5	Score 6
Score 1	-	0.45 **	0.56 **	0.38 **	0.23 **	0.40 **
Score 2		-	0.66 **	0.29 **	0.32 **	0.39 **
Score 3			-	0.35 **	0.28 **	0.43 **
Score 4				-	0.59 **	0.78 **
Score 5					-	0.60 **

** p<0.01.

**Table 5:** Correlations between information reduction and learning performance.

Information Reduction	Score 1	Score 2	Score 3	Score 4	Score 5	Score 6	Sum
Exploration Efficiency	0.20 **	0.20 **	0.27 **	0.24 **	0.24 **	0.33 **	0.34 **
Problem representation	0.24 **	0.25 **	0.32 **	0.26 **	0.28 **	0.33 **	0.38 **

** p<0.01.

**Table 6:** Distribution of three groups in three information-reduction strategies.

	Not Following Default Order	Prioritizing Respiratory Rate	Ignoring Body Temperature
High	121 (39.93%)	37 (12.21%)	67 (22.11%)
Medium	94 (31.02%)	77 (25.41%)	80 (26.40%)
Low	88 (29.05%)	189 (62.38%)	156 (51.49%)

**Table 7:** Distribution of participants across the three information-reduction strategy groups based on exploration efficiency and problem representation groups.

		High	Medium	Low	High	Medium	Low
Not following default order	High	44 (36.36%)	59 (48.76%)	18 (14.88%)	47 (38.84%)	56 (46.28%)	18 (14.88%)
	Medium	20 (21.28%)	49 (52.12%)	25 (26.60%)	20 (21.28%)	42 (44.68%)	32 (34.04%)
	Low	16 (18.18%)	35 (39.77%)	37 (42.05%)	14 (15.91%)	44 (50.00%)	30 (34.09%)
Prioritizing respiratory rate	High	16 (43.24%)	18 (48.65%)	3 (8.11%)	18 (48.65%)	15 (40.54%)	4 (10.81%)
	Medium	31 (40.26%)	37 (48.05%)	9 (11.69%)	30 (38.96%)	34 (44.16%)	13 (16.88%)
	Low	33 (17.46%)	88 (46.56%)	68 (35.98%)	33 (17.46%)	93 (49.21%)	63 (33.33%)
Ignoring body temperature	High	28 (41.79%)	34 (50.75%)	5 (7.46%)	32 (47.76%)	27 (40.30%)	8 (11.94%)
	Medium	27 (33.75%)	43 (53.75%)	10 (12.50%)	26 (32.50%)	40 (50.00%)	14 (17.50%)
	Low	25 (16.02%)	66 (42.31%)	65 (41.67%)	23 (14.74%)	75 (48.08%)	58 (37.18%)

## Data Availability

The data presented in this study are available upon request from the corresponding author.

## Note

1 (the LMC tool is implemented through web programming, which can theoretically run in any computer environment with a browser and Internet. Accessing it requires obtaining a test account, so any researcher who wants to use it can contact the corresponding author. It should be noted that LMC currently only supports the Chinese version. We welcome scholars from various language backgrounds to jointly develop versions suitable for their language and culture).

## Supplementary Materials

The following supporting information can be downloaded at https://www.mdpi.com/article/10.3390/jintelligence13030028/s1, Personal Information and Task Experience Questionnaire, and Anonymized Data.

## Abbreviations

The following abbreviations are used in this manuscript:

LMC	Little Monster Clinic
CPS	complex problem-solving
LSE	linear structural equation
FSA	finite state automata
