# Supplementary material for: Information-Reduction Ability Assessment in the Context of Complex Problem-Solving
Source: J Intell. 2025 Feb 26;13(3):28. doi: 10.3390/jintelligence13030028 (PMC11942883; doi:10.3390/jintelligence13030028)
Supplement: Supplementary file 1 [file jintelligence-13-00028-s001.zip › Supplementary Material - Questionnaire.pdf]

Supplementary Material

Personal Information and Task Experience Questionnaire

This questionnaire consists of 21 questions, each pertaining to your basic information or your experience with the task. There are no "right" or "wrong" answers; please respond according to your true situation or thoughts. Your feedback is very important to us, and we appreciate your careful responses.

1. School:

2. Grade:

3. ID:

4. Age:

5. Gender:

6. Father's occupation:

    A. Healthcare worker

    B. Non-healthcare worker

7. Mother's occupation:

    A. Healthcare worker

    B. Non-healthcare worker

8. Frequency of hospital visits due to illness:

    A. Less than once a year

B. 1-3 times a year

C. 3-5 times a year

D. 5-7 times a year

E. More than 7 times a year

9. Time spent playing computer games:

A. Almost never

B. Less than 3 hours per week

C. 3-5 hours per week

D. 5-10 hours per week

E. More than 10 hours per week

10. The operation of the task is simple:

A. Strongly Disagree

B. Somewhat Disagree

C. Uncertain

D. Somewhat Agree

E. Strongly Agree

11. The tasks are challenging:

A. Strongly Disagree

B. Somewhat Disagree

C. Uncertain

D. Somewhat Agree

E. Strongly Agree

12. Introduction of the task is clear and concise:

    A. Strongly Disagree

    B. Somewhat Disagree

    C. Uncertain

    D. Somewhat Agree

    E. Strongly Agree

13. The visual design of the task is aesthetically pleasing:

    A. Strongly Disagree

    B. Somewhat Disagree

    C. Uncertain

    D. Somewhat Agree

    E. Strongly Agree

14. The task design can arouse my interest:

    A. Strongly Disagree

    B. Somewhat Disagree

    C. Uncertain

D. Somewhat Agree

E. Strongly Agree

15. The process of the task makes me feel happy:

A. Strongly Disagree

B. Somewhat Disagree

C. Uncertain

D. Somewhat Agree

E. Strongly Agree

16. Completing task gives me a sense of achievement:

A. Strongly Disagree

B. Somewhat Disagree

C. Uncertain

D. Somewhat Agree

E. Strongly Agree

17. The task reflects my problem-solving ability:

A. Strongly Disagree

B. Somewhat Disagree

C. Uncertain

D. Somewhat Agree

E. Strongly Agree

18. The task has a certain level of difficulty:

A. Strongly Disagree

B. Somewhat Disagree

C. Uncertain

D. Somewhat Agree

E. Strongly Agree

19. The task requires my full attention and careful thought:

A. Strongly Disagree

B. Somewhat Disagree

C. Uncertain

D. Somewhat Agree

E. Strongly Agree

20. Which of the following medications are antibiotics? (Select all that apply)

A. Penicillin

B. Cephalosporin

C. Norfloxacin

D. Morphine

E. Paracetamol

F. Chlorpheniramine

G. Diazepam

H. Huoxiang Zhengqi Water

I. 999 Cold Remedy

J. Yinqiao Detoxification Tablets

K. Amoxicillin

21. Which of the following medications require a prescription? (Select all that apply)

A. Penicillin

B. Cephalosporin

C. Norfloxacin

D. Morphine

E. Paracetamol

F. Chlorpheniramine

G. Diazepam

H. Huoxiang Zhengqi Water

I. 999 Cold Remedy

J. Yinqiao Detoxification Tablets

K. Amoxicillin
